# Left ventricular pseudoaneurysm after ascending, arch and descending aortic replacement with Evita Open Plus® prosthesis

**DOI:** 10.1186/1749-8090-8-S1-P2

**Published:** 2013-09-11

**Authors:** JR Echevarría, M Fernández, M Blanco, H Valenzuela, G Laguna, P Pelaez

**Affiliations:** 1 Hospital Clínico, Valladolid, Spain

## Background

We report a rare complication after successful Evita Open Plus® implantation with cardiopulmonary bypass and circulatory arrest complete for total aortic aneurysm treatment.

## Methods

A 71 year old male with aortic aneurysm was referred for consideration of surgery. The patient comes to the emergency department to present an acute left chest pain. He was a history of duodenal ulcer and arterial hypertension. Rx thorax demonstrated mediastinal widening compatible with giant aortic aneurysm. The echocardiogram (TTE) confirmed normal sized cardiac cavities and ascending aortic aneurysm. Aortography presented severe dilatation of ascending thoracic aorta with moderated aortic regurgitation. CT Angiography established supra-aortic ascending aneurysm with maximum dimension of 53 mm, 65 mm arch aneurysm and descending 106 mm in the transverse plane.

Upper medial resternotomy, cardiopulmonary bypass and circulatory arrest complete with moderated hypothermia were performed as well as ascending aorta and arch replacement with reimplantation of the supra-aortic trunks by hybrid prosthetic Evita Open Plus® 33.

## Results

The patient evolution was favourable. In the control TTE was observed on the left ventricular apex two interconnected cavities support with left ventricular pseudoaneurysm, confirmed by CT angiogram. The patient required a new intervention, performing exclusion of the pseudoaneurysm in the left ventricular (LV) apex with a Teflon patch. After surgery remains stable and was discharged without complications.

## Conclusion

The total aortic replacement (ascending aorta, arch and descending aorta) using hybrid routines are reserved for patients with complex pathology. Iatrogenic LV pseudoaneurysms postoperative may uncommon. In some cases is related to the increase in consistency (due to hypothermia during circulatory arrest complete) of the cannula vent of the LV during extracorporeal circulation.

